# Virtual Extensometer Analysis of Martensite Band Nucleation, Growth, and Strain Softening in Pseudoelastic NiTi Subjected to Different Load Cases

**DOI:** 10.3390/ma11081458

**Published:** 2018-08-17

**Authors:** Cagatay Elibol, Martin F.-X. Wagner

**Affiliations:** 1 Institute of Materials Science and Engineering, Chemnitz University of Technology, D-09107 Chemnitz, Germany; martin.wagner@mb.tu-chemnitz.de; 2 Department of Materials Science and Technology, Turkish-German University, 34820 Istanbul, Turkey

**Keywords:** deformation/strain analysis, digital image correlation (DIC), NiTi shape memory alloys, stress-induced martensitic transformation, virtual extensometer

## Abstract

Pseudoelastic NiTi shape memory alloys exhibit different stress–strain curves and modes of deformation in tension vs. compression. We have recently shown that under a combination of compression and shear, heterogeneous deformation can occur. In the present study, we use digital image correlation to systematically analyze how characteristic features of the nominally uniaxial engineering stress–strain curves (particularly the martensite nucleation peak and the plateau length) are affected by extensometer parameters in tension, compression, and the novel load case of shear-compression. By post-experimental analysis of full surface strain field data, the effect of the placement of various virtual extensometers at different locations (with respect to the nucleation site of martensite bands or inhomogeneously deforming regions) and with different gauge lengths is documented. By positioning an extensometer directly on the region corresponding to the nucleating martensite band, we, for the first time, directly record the strain-softening nature of the material—a specific softening behavior that is, for instance, important for the modeling community. Our results show that the stress–strain curves, which are often used as a basis for constitutive modeling, are affected considerably by the choice of extensometer, particularly under tensile loading, that leads to a distinct mode of localized deformation/transformation. Under compression-shear loading, inhomogeneous deformation (without lateral growth of martensite bands) is observed. The effects of extensometer gauge length are thus less pronounced than in tension, yet systematic—they are rationalized by considering the relative impact of differently deforming regions.

## 1. Introduction

NiTi shape memory alloys (SMAs) exhibit two unusual characteristics: shape memory and pseudoelasticity. The shape memory effect refers to the ability of an SMA to reduce large, mechanically induced strains (in the low temperature phase, martensite) and to recover its original shape by post-deformation heating above a transition temperature (which transforms the material into the high temperature phase, austenite). Pseudoelasticity describes a shape memory material`s ability to return to an initial shape during unloading after considerable deformation (with uniaxial transformation strains in polycrystalline NiTi of about 6%) at a constant temperature. The underlying microstructural mechanisms for this material behavior primarily result from the reversible, stress-induced martensitic transformation (SIMT) from austenite to martensite during loading, and its reversal during unloading at a sufficiently high ambient temperature. NiTi alloys are the most important SMAs with outstanding pseudoelasticity and good fatigue resistance, and are frequently used in commercial applications, such as medical devices [1,2,3,4,5,6].

It is well-known that during pseudoelastic deformation of NiTi, the transformation occurs in a localized manner (i.e., via nucleation and propagation of well-defined martensite bands) under uniaxial tension [7,8,9,10]. Because of this localized mode of deformation, local and nominal stresses and strains differ considerably. The results of finite element simulations, such as using a total deformation strain model [11] capable of modeling the non-linear mechanical behavior of NiTi SMAs, show that an implementation of material softening allows simulation of the inhomogeneous, stress-induced martensitic phase transformation in NiTi SMAs under tension. In these models (e.g., see also [9,12,13,14]), the nucleation stress of the austenite to martensite transformation is higher than the stress required to continue the SIMT. Direct experimental proof of material softening, however, is challenging as band propagation occurs at a constant stress rate and the nominal stress–strain curves typically exhibit a constant stress plateau. One goal of this study was to contribute to a better understanding of the softening nature of pseudoelastic NiTi (and thus supporting numerical modeling approaches that incorporate material softening) by providing additional experimental observations of this phenomenon.

The mechanical response of NiTi subjected to simple compressive loading is quite different [7,8,15,16,17]. While a constant stress plateau in the stress–strain curves is clearly associated with the stable propagation of martensite bands in tension, the macroscopic deformation of polycrystalline SMAs in compression proceeds homogeneously, and no flat stress plateau is observed [18]. The tension compression asymmetry and the pseudoelastic hysteresis led to interesting strain/phase distributions under more complex load cases, such as bending [19,20,21], bending rotation [3,22], or combinations of torsion and tension [12,23,24]. In recent works [7,8], we have also demonstrated that localized deformation is not necessarily limited to tensile loading alone—compression-shear loading (i.e., a combination of predominantly compressive loading with a certain amount of superimposed shear stresses) can also lead to a localization/inhomogeneous distribution of the SIMT. These studies have highlighted that a careful analysis of local strain fields is required to fully characterize the complex material response of NiTi SMAs to these different load cases. Compression-shear loading in particular has not been studied in-depth before, and there is a need to relate local and global strains in order to accurately determine representative stress–strain data for this novel experimental load case.

In this study, the in-situ optical technique digital image correlation (DIC) was used to measure the surface strain fields during quasi-static uniaxial tension, compression, and compression-shear loading. Special emphasis was placed on the effect of the gauge length and positioning of “virtual extensometers” (which can be defined after an experiment based on the full-field DIC data) on the nominal mechanical behavior of a pseudoelastic NiTi bar material. We discuss the relationship between the macroscopic deformation mode, the positioning of a virtual extensometer with respect to the nucleation site of a martensite band, and their effects on distinct features of the resulting stress–strain curves (especially on the shape of the nucleation peak that is typically observed prior to the stress plateau under tension, and on the macroscopic transformation strains). As the most interesting special case, we also discuss the strain-softening behavior of the material by analyzing the deformation in the distinct region of martensite band nucleation. Our results confirm that the macroscopic stress–strain curves, which are often used as a basis for constitutive modeling without considering important experimental details, can be affected considerably by the positioning of extensometers, particularly under tensile loading, and that the deformation mode of compression-shear tests differs significantly from the homogeneous or distinctly localized modes of compression and tensile testing, respectively.

## 2. Materials and Methods

A commercial polycrystalline NiTi bar (diameter 12.7 mm) with a nominal alloy composition of 50.9 at. % Ni (obtained from Memry Corp., Bethel, CT, USA) was used in the experiments. All specimens for mechanical testing were annealed at 623 K for 3.6 ks, followed by a water-quench to room temperature. This heat treatment led to the formation of Ni_4_Ti_3_ precipitates [25] and resulted in a higher critical stress level for irreversible plastic deformation by a dislocation slip, thus improving pseudoelastic behavior of the NiTi material. During this heat treatment, the average grain size remained constant (~45 µm) and the material showed excellent pseudoelastic recovery [7,8]. Further details on microstructural characterization of the as-received and heat-treated materials are presented in [7].

**Figure 1:**
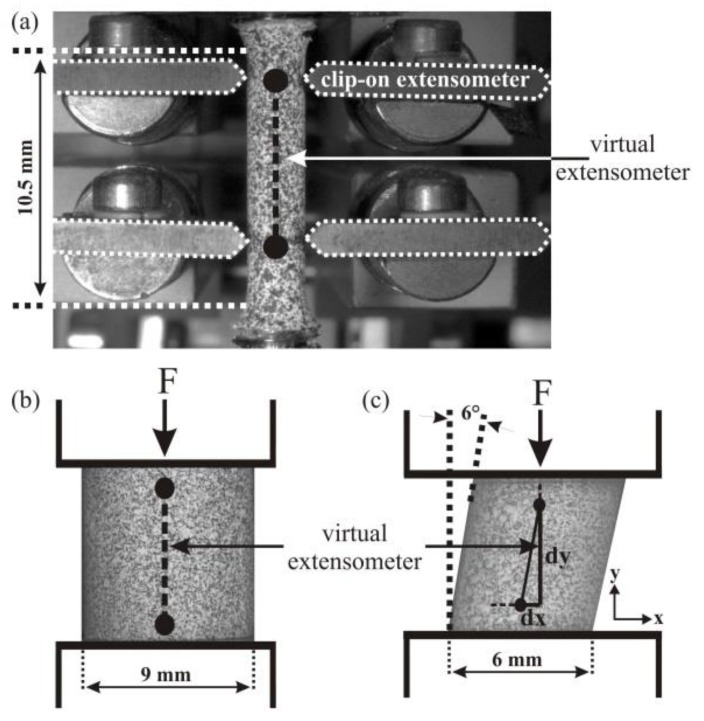
Geometries of the samples used for the mechanical testing and artificially-produced speckle patterns on the samples required for the measurement of the surface strain fields using digital image correlation (DIC): (**a**) cylindrical dog-bone-shaped tensile test sample; (**b**) cylindrical compression test sample; (**c**) cylindrical compression-shear test sample with an inclination angle of 6° that results in superimposed shear stresses during compressive loading. The conventional clip-on extensometer for tensile testing (indicated in (**a**)) and virtual extensometers used for the deformation measurements in all our mechanical tests are shown schematically on the specimen surfaces. In tensile, compression, and also in compression-shear tests, the uniaxial strain was determined from the DIC images by considering two surface points (black dots) and their relative displacements in the axial direction, *dy*; positioning of these points was varied in post-experimental analysis.

In this study, all tension, compression, and compression-shear experiments were conducted at room temperature under slow, quasi-static loading conditions (at a strain rate of 10^−4^ s^−1^), which is important to a reduce the heat effect caused by the latent heat of the stress-induced martensitic transformation on the thermomechanical response of pseudoelastic NiTi [8,26]. The uniaxial tensile tests were performed on a conventional tensile/compressive testing machine (Zwick Allround-Line 20 kN, Ulm, Germany) under displacement-controlled loading conditions. Dog-bone-shaped specimens with a free gauge length of 10.5 mm and a diameter of 3.56 mm were tested (Figure 1a). The uniaxial compressive and combined compression-shear tests were performed in a universal testing machine (Zwick UPM1475-100 kN, Ulm, Germany). For simple compression testing, cylindrical specimens with a diameter of 9 mm and a length of 9 mm (the short aspect ratio prevents bending and buckling that can easily occur in NiTi because of the large strains associated with the SIMT) were used, and for combined compression-shear tests, cylindrical samples (6 mm diameter × 6 mm length) with an inclination angle of 6° with respect to the loading direction, were used (see Figure 1b,c).

### 2.1. Optical Strain Measurements Using DIC

Using the in-situ optical DIC technique, the surface strain fields (i.e., the deformation on the surface of the specimens) were measured in all experiments. The non-contact DIC method provided full-field measurements of surface displacements and strains [27,28,29] and provided complementary information to e.g. diffraction experiments, where phase volume fractions in the surface or in the bulk of a sample could be determined [30,31,32]. During the deformation, the spatial coordinates of the specimen surface were changed. The DIC software (v6.3.1) first computed the displacements of each point (pixel) on the surface of the sample by comparing the digital images recorded at different times and then determined the local surface strain fields from these displacements. For deformation measurements, a random gray intensity distribution on the sample surfaces was therefore required so that the random speckle patterns could be tracked [33]. In our experiments, an artificially-applied pattern was produced by spraying white and black paint using air-brush equipment onto the specimen surfaces (see also Figure 1). DIC images were captured with a frame rate of 1 Hz using a single CCD camera (with a resolution of 2358 × 1728 pixels) that was placed perpendicularly to the specimen surfaces. The optical deformation analysis (ARAMIS) software package (v6.3.1) [34] was used for analysis of the DIC images; the combination of the optical system (lenses), detector (pixel), and the software determined the resolution. In the experiments presented here, strain values that can be allocated to a pixel width of approx. 10 µm (~10 µm/pixel) could be determined. 

### 2.2. Determination of Nominal Uniaxial Stresses and Strains

In uniaxial tensile and compressive experiments, the engineering stress can simply be defined as the tensile/compressive force divided by the initial cross-sectional area of the specimen. Under combined compression-shear loading, however, the deformation is not entirely uniaxial and the corresponding stress state is somewhat more complex. While we have recently performed numerical investigations on stresses and strains during the early, elastic loading of compression-shear specimens [35], in the present study (where the SIMT dominates the material behavior once a critical stress is reached) we only consider nominal compressive stresses and strains—i.e., load and deformation components in the compression direction were used to calculate representative, “nominal” stress and strain values.

For the determination of uniaxial engineering strains from the DIC data, in particular, two points on the DIC image of the specimen surface were considered in the non-deformed state (see also black dots in Figure 1). During the uniaxial (tension or compression) experiments, the relative displacements of these points in axial deformation direction were recorded. The engineering strains were then calculated from the axial displacements, considering the initial distance between these two points. Likewise, under compression-shear loading, nominal strains were determined by considering only the axial component of the displacements, *dy*, but neglecting changes in the transverse direction, *dx*. Using nominal stress and strain values, we determined representative stress–strain curves that could be directly compared to, and analyzed in light of, the corresponding data from tension and compression experiments.

The method of strain determination by tracking the axial displacements of two points can be considered as using a virtual extensometer, which is quite similar to the macroscopic strain measurement using conventional clip-on extensometers (see, e.g., Figure 1a). A previous study [36] demonstrated that using the DIC virtual extensometer method was just as reliable as clip-on extensometers typically used during tensile testing to determine engineering strains in NiTi SMAs. Indeed, deformation measurements using virtual extensometers are even more accurate than those performed with inductive calipers (typically used in compression testing), because when conventional inductive calipers are used with small compression samples, the deformation often cannot be measured directly on the specimens—the calipers instead need to be attached to adjacent parts of the experimental setup, and the displacement signal thus contains information about the deformation that is not limited to the sample itself. Moreover, as, locally, even small increases in stress (which may result from the application of an extensometer) can trigger the SIMT (e.g. in thin wire or ribbon samples), DIC methods provide an additional advantage since they are entirely contact-free. Finally, the DIC method also provides much more information about local deformations on the specimen surface in complex load cases (which is particularly relevant when analyzing compression-shear loading). An in-depth analysis of local strain fields for the three experiments considered in the present study is not presented here but can be found in [7,8]. The key goal of this study was to analyze how the nominal stress–strain curves, determined using the virtual extensometer method on the different samples, are affected by the placement of the extensometers at different locations and with different gauge lengths, and particularly how the specific softening behavior can be experimentally characterized by the deliberate positioning of a virtual extensometer directly onto the region corresponding to the nucleating martensite band during uniaxial tensile loading.

## 3. Results and Discussion

In the case of localized deformation, the length/position of an extensometer has a significant influence on the shape of the macroscopic stress–strain curves [37]. This is often not recognized by the modeling community, where researchers simply take stress–strain data from literature and assume that these data represent homogeneous material behavior. The DIC technique provides an ideal opportunity to investigate the effect of the extensometer’s position on the stress–strain curves by simultaneous analysis of various virtual extensometers using one set of experimental data. For the purposes of such an analysis, tensile, compression, and compression-shear test samples were investigated. While we have discussed in [7,8] that these different tests are associated with different modes of deformation, the present study analyzes how the different types of tests differ in terms of their sensitivity to extensometer placement, and how these effects lead to significant variations in the macroscopic stress–strain data.

**Figure 2:**
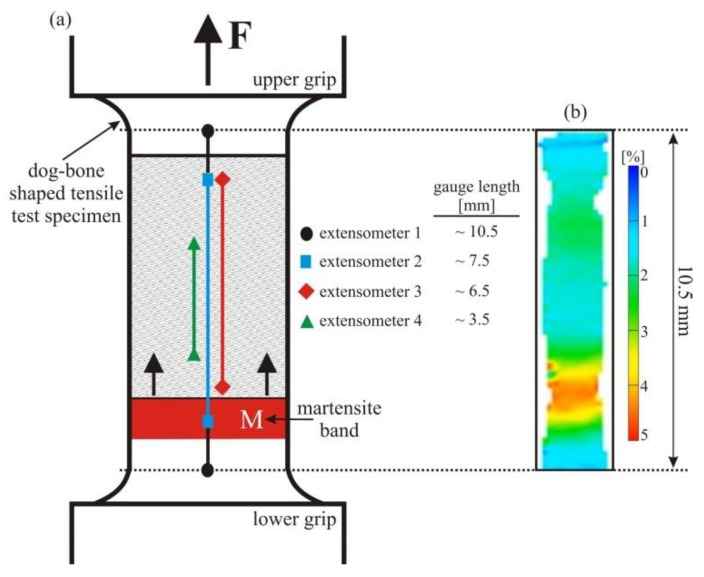
(**a**) Schematic representation of the dog-bone-shaped tensile test specimen with four virtual extensometers used for investigation of the effect of the extensometer position, with respect to the nucleation side of the martensite band; (**b**) axial surface strain field image measured at a macroscopic strain of about 2.1%. The analyzed extensometers are shifted sideways from the center line (where they were located in the actual analysis of DIC data) to provide a better overview.

**Figure 3:**
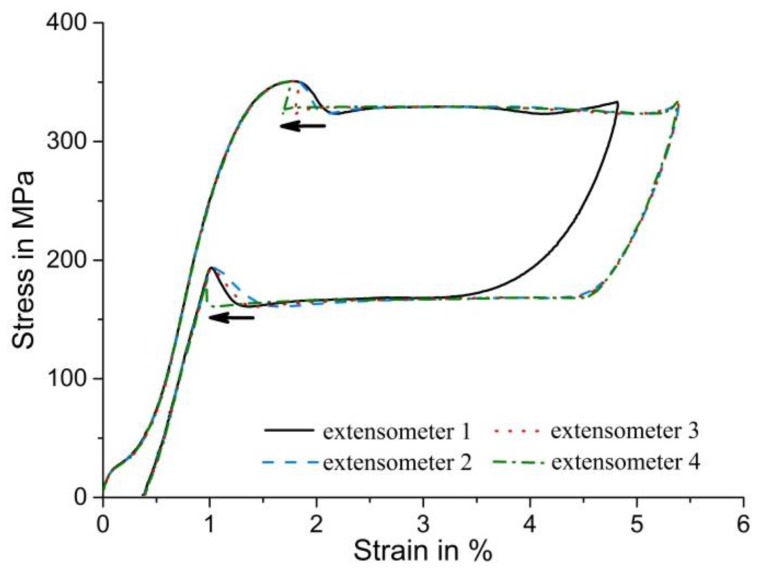
Engineering stress–strain curves determined using various virtual extensometers (see Figure 2) under uniaxial quasi-static tensile loading. The different extensometer positions with respect to the nucleation site of the martensite band lead to a systematic and significant effect on the resulting stress–strain curves, especially on the shape of the nucleation peak.

First, the effect of the extensometer gauge length was investigated under tension where the pseudoelastic NiTi SMAs exhibit a distinct mode of localized deformation (formation of martensite bands). The positions of the various virtual extensometers analyzed in the same experiment, and the martensite band (M) formed during the deformation on reaching a critical transformation stress near the lower grip, are shown schematically in Figure 2a. The four analyzed virtual extensometers were all located centrally along the longitudinal axis of the dog-bone-shaped tensile test specimen. During tensile experiments at low strain rates, a single martensite band was nucleated, which grew along the gauge length of the sample. The propagation of the interface of the distinct martensite band proceeded at a constant tensile stress rate, leading to a flat stress plateau in the macroscopic stress–strain curve (see also Figure 3) [7,9,15,26,38,39]. A typical surface strain field, recorded during propagation of the band at a macroscopic strain of about 2.1%, is shown in Figure 2b. We highlight that, while similar strain field observations have been published before, and in some cases with higher magnification [19,27], in the present study our primary aim was to simply visualize the different modes of deformation in tension, compression, and shear-compression; the resolution of the strain maps presented here is obviously sufficient to achieve this goal. 

**Figure 4:**
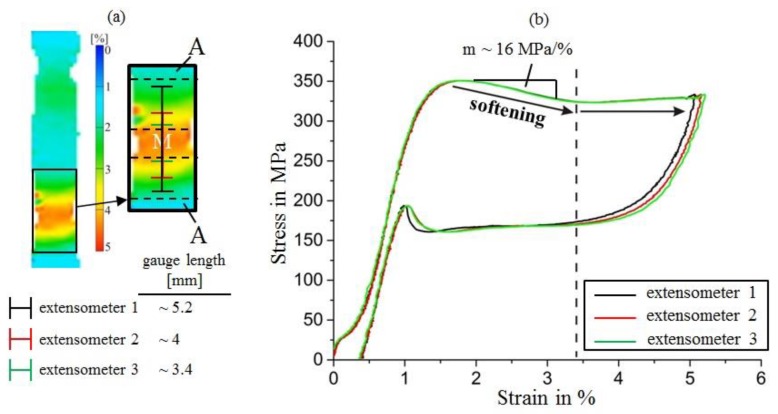
(**a**) Placement of several virtual extensometers in the region where martensite band nucleation first occurs. The strain map clearly shows that (in contrast to the grown band during latter stages of deformation), lower strains occur because the band’s nucleus effectively consists of two meso-scale interfaces that have not yet moved apart; (**b**) Effect of positioning the virtual extensometers in direct proximity to the martensite band on the resulting engineering stress–strain curves during tensile loading. For all virtual extensometers located in the region of the nucleating martensite band, broad nucleation peaks that exhibit pronounced softening are observed.

Prior to analyzing the effect of different virtual extensometers on the stress–strain curves, it is important to note that, while the martensite band grows, all deformation occurs only at the meso-scale “interface” with a finite width between the (still) fully austenitic and the (already) fully martensitic regions [7,9,10,30]. These meso-scale interfaces represent transition regions (see also Figure 4a) that span the entire width of a specimen, where phase fractions of martensite and austenite, and stress and strain fields change significantly. In the martensite band itself, and in the austenitic regions further away from the meso-scale interface, no deformation takes place. Moreover, it is well-known that the initial formation of the martensite band requires higher stress levels than subsequent propagations of the meso-scale phase interface; this typically leads to a “nucleation peak” and a subsequent stress-drop to the plateau stress level in the macroscopic stress–strain curve of pseudoelastic NiTi [38,40,41]. It is thus to be expected that the positioning of an extensometer relative to the location of initial martensite formation directly affects the macroscopic stress–strain curve. We note in passing that nucleation peaks are most pronounced at low strain rates, whereas samples of self-heating related to the SIMT (and the corresponding increase of the critical stress level for the SIMT) at higher rates tend to mask nucleation peaks [7,8]; the present study is therefore focused on slow, quasi-static experiments.

Figure 3 shows the effect of the various extensometers (positions defined in Figure 2) on the resulting stress–strain curves during tensile loading. In the early stages of apparently elastic loading, non-linear behavior takes place at very low stress points. This effect is associated with the stress-induced formation of an intermediate, martensitic R-phase; further information on this phenomenon, which is not investigated further in the present paper, is given, e.g., in [42]. Two main observations that are discussed below concern the absolute length of the pseudoelastic plateau and the shape of the nucleation peak. It is clear from Figure 3 that the plateau length (measured from the strain at maximum nucleation peak stress) is very similar for extensometers 2, 3, and 4. Despite the different lengths/positions, all of these extensometers provide the same value of total strain, e.g., at the end of the plateau when the SIMT is completed throughout the specimen. The length of the plateau represents the (uniaxial) transformation strain associated with the SIMT, but elastic strain components (which are different in austenite and martensite because of different Young’s moduli) need to be carefully subtracted to arrive at physically reasonable transformation strain values. The martensitic phase transformation thus produces a characteristic macroscopic strain, and because from the beginning to the end of the transformation, the martensite band is formed and then grows entirely within the gauge lengths of extensometers 2–4, once the transformation is complete, all extensometers are attached to fully martensitic material and must measure a similar macroscopic strain value. The longer extensometer 1, in contrast, produces strain data that leads to a shorter length of the stress plateau (and therefore to lower total strain values at any given time during the experiment; the difference in total strain is about 0.5%). This result indicates that the deformation at the ends of the parallel length of the sample (where the two extensometer points associated with extensometer 1 were placed) is considerably constrained by the adjacent regions that, because of the increasing cross-section, deform less. While this effect is typically negligible when analyzing homogeneous deformation, the impact on total strains is much more pronounced here—in the regions with larger cross-sections, true stresses decrease rapidly and no SIMT takes place. Therefore, while the complete uniaxial transformation strain contributes to the sample deformation up to more than 5% inside the parallel length of the sample, the adjacent regions still consist of (elastically strained) austenite, and thus contribute much less to the nominal uniaxial strain measured by extensometer 1.

The second and more important observation is that the shape of the nucleation peak at the beginning of the pseudoelastic stress plateau changes considerably depending on the position of the virtual extensometers. Figure 3 clearly shows that the macroscopic strain measured by extensometers 1 and 2 increases during the stress-drop process at the beginning of the stress plateau. This is because the nucleation stage of the martensite band (during which the strain increases locally) occurs within both gauge lengths. In stark contrast, the strain data from extensometers 3 and 4 indicate that strains in the sample actually decrease during the nucleation of the martensite band—an observation that, at first glance, seems to contradict the experimental procedure (where a constant displacement rate was imposed on one end of the sample). This discrepancy can be rationalized by considering the relative position of extensometers 3 and 4 with respect to the nucleation site—the martensite band was formed near, but outside, the gauge length of extensometer 3. Local nucleation of a martensite band leads to a spontaneous elongation and thus to a stress-drop—obviously, the adjacent, austenitic regions are temporarily elastically unloaded. This effect was recorded by extensometer 3: Until its gauge region was reached by the growing martensite band, the extensometer only covered areas that were first elastically unloaded and then remained static (i.e., elastically deformed under a constant stress). The gauge length of the shorter extensometer 4 was reached only later by the propagating martensite band, compared to extensometer 3. This systematic effect of extensometer location with respect to the nucleation site on the nucleation peak shape is highlighted by the black arrow in Figure 3. A similar phenomenon is observed at the end of the stress plateau during unloading: During the reverse transformation from martensite to austenite, the martensite band shrinks and the final martensitic region becomes unstable. It suddenly collapses to form austenite, which leads to a local contraction in axial direction and thus to a temporary increase of uniaxial stress. Again, the corresponding stress peaks in the stress–strain curves are systematically shifted depending on whether the localized event occurred inside (extensometers 1 and 2) or outside (extensometers 3 and 4) the respective virtual gauge lengths. In summary, these results clearly show that, in addition to the total strain measured, the shape of the nucleation/transformation completion peaks can also be significantly and systematically changed by modifying the positions and gauge lengths of the virtual extensometers when evaluating tensile experiments on pseudoelastic NiTi. Despite the fact that the four stress–strain curves represent the same experiment, they might, without proper consideration of the effect of extensometer positioning, be interpreted quite differently (e.g., in terms of broad vs. sharp nucleation peaks, and large vs. reduced transformation strains).

Although stress–strain curves, determined experimentally, typically provide information about macroscopic mechanical behaviors, for numerical modeling, the local deformation characteristics (e.g., under uniaxial tensile loading) need to be captured. Discrepancies arise whenever localized deformation occurs, as is clearly the case in the pseudoelastic NiTi material studied here. The observation of nucleation peaks shows that material softening is associated with martensite band nucleation, and as growth of the band corresponds to propagation of meso-scale interfaces, similar softening occurs locally as long as the transformation is not completed along the entire gauge length. Our observations of the effect of different extensometer gauge lengths on the shape of the nucleation peak inspired us to specifically analyze the nucleation event exclusively in the region where a martensite band is formed. Virtual extensometers were thus deliberately located directly on (and covering only) the region where the DIC data showed nucleation of the martensite band to occur. Figure 4a shows this region at higher magnification, including a schematic representation of the virtual extensometers (the dashed lines represent the approximate boundaries of this region). As clearly outlined in Figure 4b, the positioning of the virtual extensometers directly onto the region corresponding to the forming martensite band leads to a much broader nucleation peak in the corresponding stress–strain curves, supporting the theoretical interpretation that strain-softening is used for modeling the localized deformation/transformation in NiTi SMAs. A previous study [43] made use of an ingenious experiment combining layers of (work-hardening) steel with a layer of NiTi to evaluate the softening slope of the NiTi stress–strain curve. Our experimental investigations, to the best of our knowledge, demonstrate for the first time that this softening behavior can also be directly analyzed experimentally in uniaxial tension in a monomaterial sample.

The slope of the softening part of the stress–strain curves, which can be considered as a very broad nucleation peak, is approximately 16 MPa/%, which is substantially different to the nearly constant stress level typically observed in the nominal stress–strain curves of pseudoelastic NiTi. This corresponds to a stress-drop of about 50 MPa over a strain range of about 3%, as shown in Figure 4b. These values may inform future modeling studies that aim at capturing localization phenomena in NiTi. 

The principal idea of this experiment was to directly capture strain softening in NiTi. We initially expected the resulting stress–strain curves to exhibit softening along the entire transformation region up to uniaxial strains of about 5%, similar to the analysis presented in [43] (where an even more pronounced softening of about 30 MPa/% was reported in NiTi sheet material). Instead, the softening/nucleation peak measured by all extensometers ended at a surprisingly similar macroscopic strain value of approx. 3.4%, and the stress remained constant at higher strains. Closer analysis of the DIC data provides an explanation for this—the macroscopic stress drop occurs during nucleation of the martensite bands, and this process corresponds to the formation of two meso-scale interfaces (with surface strains between about 1.5 and 4.5%). Only once these interfaces move apart (which occurs at constant stress as the same process, the passing of an interface across yet untransformed material, occurs repeatedly), the martensite band begins to grow and the fully transformed martensitic region in the center of the band can be observed. It is important to understand that, in agreement with the data presented in [43], material softening is likely to occur until the SIMT is completed, but the local reduction of stress is related to the complex stress state in the moving interfaces [44], whereas only the initial stress drop during band nucleation could be measured macroscopically and related to the intermediate strains observed in the interface regions in our experiments. 

**Figure 5:**
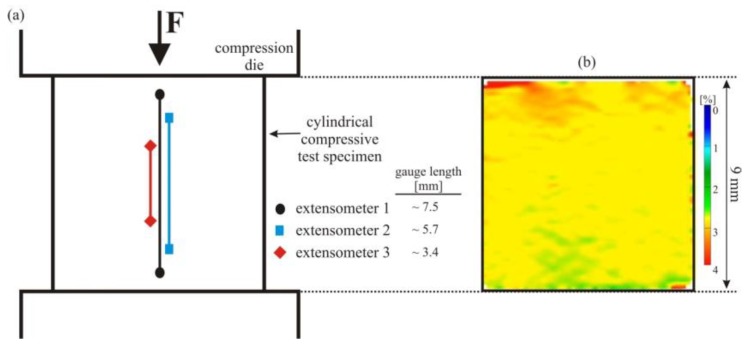
(**a**) Schematic representation of the cylindrical compressive test specimen; (**b**) axial surface strain field recorded at a macroscopic strain of about 3%. Three virtual extensometers (again located next to each other for a better overview) were used under simple compression, where the martensitic transformation proceeds homogeneously on a macroscopic scale without distinct strain localization.

Next, we discuss the effect of the position of virtual extensometers on the resulting stress–strain curves of pseudoelastic NiTi under simple compression. Three different extensometers were used to determine uniaxial strain values. The arrangement of these extensometers, again placed centrally along the longitudinal axis of the compression specimen, is illustrated in Figure 5.

**Figure 6:**
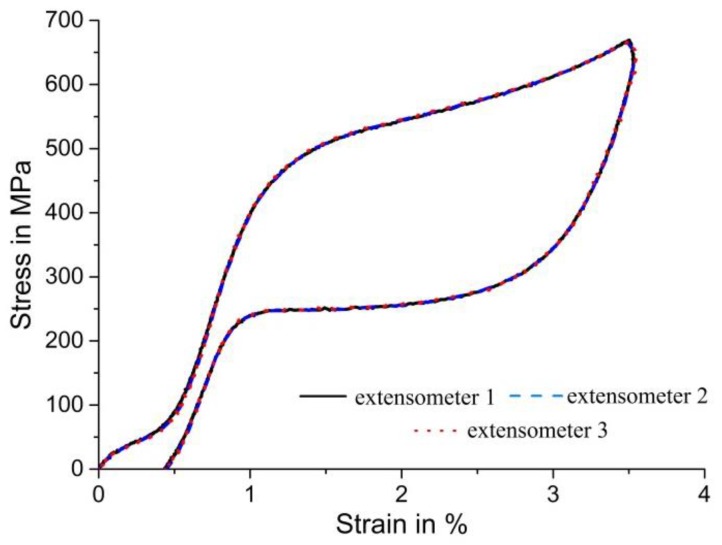
Engineering stress–strain curves determined using various virtual extensometers under simple compression. The different positions and gauge lengths of the virtual extensometers (see Figure 5) have no effect on the resulting stress–strain curves due to the very homogeneous deformation of pseudoelastic NiTi in compression.

Figure 6 shows the stress–strain curves of the pseudoelastic NiTi SMA under uniaxial compression. In contrast to tensile loading of NiTi, the transformation/deformation proceeds homogeneously and without the formation of distinct martensite bands under simple compression (see also Figure 5b). Moreover, no flat stress plateau is observed in the corresponding stress–strain curves. Pseudoelastic NiTi exhibits a well-known tension/compression asymmetry in terms of the critical stresses required for the initial formation of martensite (see also Figure 3 and Figure 6, where the stress at the onset of the SIMT is about 100 MPa higher in compression than in tension). The strong difference in the macroscopic stress–strain curves under tension and simple compression then continues as straining through the transformation region produces a “hardening” response under compression. The different macroscopic mechanical behavior is associated with highly different deformation modes [7]. As Figure 6 clearly shows, the variations of the lengths of the different extensometers have no influence on the resulting stress–strain curves—all extensometers provide the same strain value at any given time during the experiment, and all curves fall on top of each other. This result confirms that under simple compression, martensite does not form in a localized manner (by formation and growth of distinct martensite bands)—instead, the deformation proceeds very homogeneously in the entire compression specimen. We note in closing that friction at the top and bottom surfaces of cylindrical compression specimens can lead to multiaxial stress-states and inhomogeneous deformation in the top and bottom regions. These regions, however, were not covered by the three different virtual extensometers used in the present study. The exact match of the three stress–strain curves also demonstrates the impressive accuracy of DIC-based methods. 

The stress–strain curves based on virtual extensometer strains (Figure 3 and Figure 6) confirm that NiTi exhibits very different modes of deformation in tension (strongly inhomogeneous: formation and growth of martensite bands) vs. compression (homogeneous transformation/deformation). Our previous studies on the localization of the SIMT under quasi-static [7] and dynamic [8] loading conditions have further demonstrated that during compression-shear loading, the superimposed shear stresses can lead to inhomogeneous deformation, even under predominantly compressive loading. However, the martensitic regions in this case are not as sharply separated from austenitic regions as the distinct martensite bands under tensile loading, and no lateral growth of a martensite band is observed [8]. An analysis of the effect of different virtual extensometer gauge lengths may thus also help to better classify the material behavior/deformation mode in compression-shear loading.

**Figure 7:**
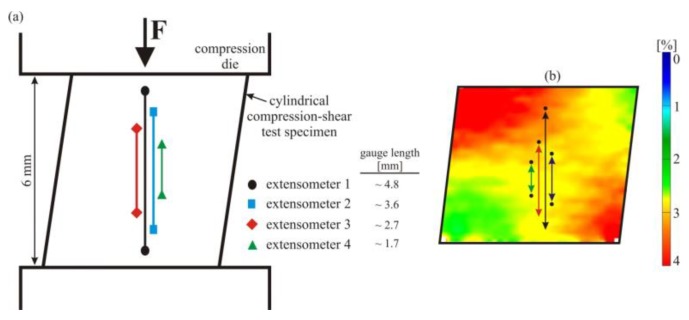
(**a**) Schematic representation of the cylindrical specimen with an inclination angle for compression-shear testing; (**b**) axial surface strain field image measured at the end of compression-shear loading (prior to unloading). Four virtual extensometers that are located here next to each other for a better overview were investigated under compression-shear loading, where the strain fields are inhomogeneous (localization of deformation) due to the more complex stress-state created by the superimposed shear stresses and the special specimen geometry.

Figure 7a schematically shows the positioning of four different virtual extensometers with different gauge lengths. The axial surface strain field image (taken at approx. 3.2% of nominal, uniaxial strain, directly prior to unloading), shown in Figure 7b, gives a detailed impression of the inhomogeneous distribution of strains during the compression-shear experiments. To ensure a better overview, the virtual extensometers analyzed were again placed side by side in the schematic representation of the compression-shear sample (left) as well as on the DIC strain field image (right), but for evaluation they were actually all located on the center line of the specimen (i.e., on top of the line given for extensometer 1). In contrast to simple compression, under compression-shear, pseudoelastic NiTi clearly exhibits a pronounced inhomogeneity of deformation that is related to the special specimen geometry and the resulting inhomogeneous stress distribution. While regions with a high martensite volume fraction (as indicated by the high axial strains) are observed next to much less strained regions, strains (and phase volume fractions) change gradually from one region to the other, and a distinct meso-scale interface region cannot be defined.

**Figure 8:**
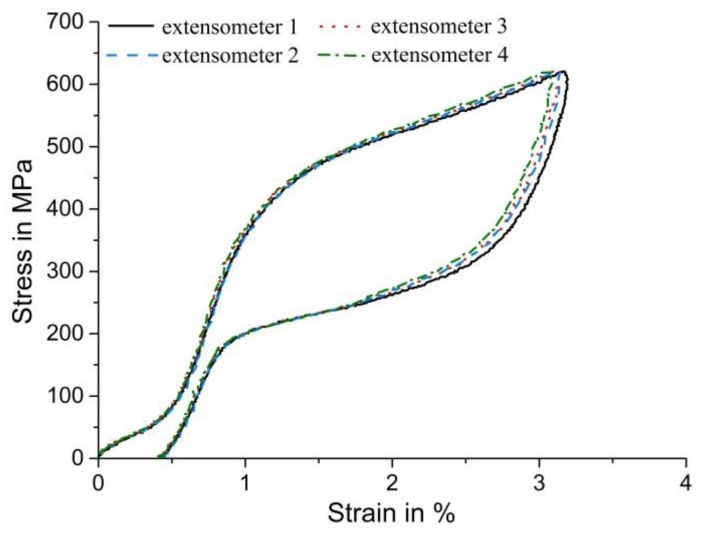
Nominal/axial engineering stress–strain curves determined under compression-shear loading. Various positions of virtual extensometers (shown in Figure 7a) result in a small but systematic effect on the resulting stress–strain curves. Larger gauge lengths are associated with larger total strain values, because they put more relative weight on the regions that exhibit the largest strains.

Clearly, the length of a virtual extensometer determines the ratio of highly strained regions vs. less strained regions considered when measuring nominal strains, and this is reflected by the corresponding stress–strain data in Figure 8. The small but systematic effect can again be directly related to the different lengths of the virtual extensometers: The longest extensometer 1 measures the highest axial deformation values because it covers a substantial part of the mostly martensitic regions (red regions in Figure 7b, representing the maximum load of the specimen). We note that those regions are the most highly strained regions throughout loading and unloading, which is why the systematic effect affects total strain values throughout the experiment. Considering extensometers 2–4, the gauge length is further reduced—hence, increasingly less information from the predominantly martensitic regions is collected. In particular, the shortest extensometer 4 only takes the deformation of the incompletely transformed regions in the center of the specimen into account, and thus records the lowest total strain values throughout the experiment. In contrast to tensile loading, the difference in strain values inside the virtual extensometer gauge lengths is much smaller in compression-shear—therefore, the effect of the extensometer position on the resulting stress–strain curves, shown in Figure 8, appears to be relatively small. Our results clearly confirm, however, that compression-shear deformation leads to a special mode of deformation with features of heterogeneous deformation (as indicated by the effect of different extensometer gauge lengths on the macroscopic stress–strain curves) that are different to homogeneous deformation (as observed in compression) even when no distinct band growth occurs, as observed in tension.

## 4. Summary and Conclusions

We have systematically investigated the effect of the gauge lengths and positions (with respect to the nucleation site of martensite bands) of various virtual extensometers on the macroscopic stress–strain curves of a pseudoelastic NiTi bar material under quasi-static uniaxial tension, uniaxial compression, and compression-shear loading. Special focus was placed on the shape of the nucleation peak typically observed prior to pseudoelastic plateau stress. As a crucial point for those such as the modeling community, we experimentally documented the strain-softening nature of the material when subjected to uniaxial tension. The digital image correlation technique was used to measure the surface strain fields and to determine nominal axial deformation values under the different stress-states. The results of our experimental investigations can be summarized as follows:

1. Under uniaxial tension, characteristics of the stress–strain curves in terms of the absolute length of the pseudoelastic plateau, and especially in terms of the shape of the nucleation peak, can be considerably affected by the position of a (virtual) extensometer with respect to the nucleation site of distinct martensite bands. In the case of a localized deformation, which primarily occurs in tension in NiTi, the positioning of the extensometer used for the strain analysis is highly important when analyzing subtle features of the resulting stress–strain curves. We show, for the first time, that the specific softening behavior of NiTi SMAs under uniaxial tensile loading can, to some extent, be directly analyzed experimentally by locating an extensometer in post-experimental DIC data analysis directly onto the region where a martensite band is nucleated. The amount of strain softening (slope of the resulting stress–strain curve: 16 MPa/%) may form an experimental basis for advanced mechanical modeling studies.
2. The mechanical response of pseudoelastic NiTi associated with the stress-induced martensitic transformation exhibits a strong difference in tension and compression. This can be attributed to the different macroscopic modes of deformation. Under tension, the stress-induced martensitic transformation proceeds in a localized manner (via formation and growth of martensite bands). Under simple compression, the deformation proceeds homogeneously (without distinct martensite bands/strain localization. Therefore, the position and total gauge length of a (virtual) extensometer has no influence on the stress–strain curves in compression.
3. In the multi-axial load case created by the special geometry of compression-shear specimens, the material exhibits a strong inhomogeneity of the transformation. However, no lateral growth of distinct martensite bands (as observed under uniaxial tension) occurs, and therefore the macroscopic stress–strain behavior closely resembles the behavior under simple compression. However, there is a small but systematic effect of the virtual extensometer’s gauge length on the resulting stress–strain curves in this special load case. While it is less pronounced when compared to tensile loading, it clearly highlights the inhomogeneous deformation mode of NiTi when subjected to compression-shear loading.

## References

[1] Otsuka K., Ren X. (2005). Physical metallurgy of Ti–Ni-based shape memory alloys. Prog. Mater. Sci..

[2] Duerig T., Pelton A., Stöckel D. (1999). An overview of nitinol medical applications. Mater. Sci. Eng. A.

[3] Wagner M.F.-X., Eggeler G. (2006). New aspects of bending rotation fatigue in ultra-fine-grained pseudo-elastic NiTi wires. Int. J. Mater. Res..

[4] Wagner M., Richter J., Frenzel J., Grönemeyer D., Eggeler G. (2004). Design of a Medical Non-Linear Drilling Device: The Influence of Twist and Wear on the Fatigue Behaviour of NiTi Wires Subjected to Bending Rotation. Mater. Werkst..

[5] Jani J.M., Leary M., Subic A., Gibson M.A. (2014). A review of shape memory alloy research, applications and opportunities. Mater. Des..

[6] Van Humbeeck J. (2001). Shape Memory Alloys: A Material and a Technology. Adv. Eng. Mater..

[7] Elibol C., Wagner M.F.-X. (2015). Investigation of the stress-induced martensitic transformation in pseudoelastic NiTi under uniaxial tension, compression and compression–shear. Mater. Sci. Eng. A.

[8] Elibol C., Wagner M.F.-X. (2015). Strain rate effects on the localization of the stress-induced martensitic transformation in pseudoelastic NiTi under uniaxial tension, compression and compression-shear. Mater. Sci. Eng. A.

[9] Shaw J.A., Kyriakides S. (1998). Initiation and propagation of localized deformation in elasto-plastic strips under uniaxial tension. Int. J. Plast..

[10] Wagner M.F.-X., Schaefer A. (2010). Macroscopic versus local strain rates during tensile testing of pseudoelastic NiTi. Scr. Mater..

[11] Azadi B., Rajapakse R.K.N.D., Maijer D.M. (2007). Multi-dimensional constitutive modeling of SMA during unstable pseudoelastic behavior. Int. J. Solids Struct..

[12] Feng P., Sun Q.P. (2006). Experimental investigation on macroscopic domain formation and evolution in polycrystalline NiTi microtubing under mechanical force. J. Mech. Phys. Solids.

[13] Grossmann C., Schaefer A., Wagner M.F.-X. (2010). A finite element study on localized deformation and functional fatigue in pseudoelastic NiTi strips. Mater. Sci. Eng. A.

[14] Šittner P., Sedlák P., Seiner H., Sedmák P., Pilch J., Delville R., Heller L., Kadeřávek L. (2018). On the coupling between martensitic transformation and plasticity in NiTi: Experiments and continuum based modelling. Prog. Mater. Sci..

[15] Orgéas L., Favier D. (1998). Stress-induced martensitic transformation of a NiTi alloy in isothermal shear, tension and compression. Acta Mater..

[16] Orgéas L., Favier D., Liu Y., Rio G., Sun Q.P. (2002). Mechanical instability of NiTi in tension, compression and shear. IUTAM Symposium on Mechanics of Martensitic Phase Transformation in Solids.

[17] Liu Y., Xie Z., van Humbeeck J., Dealey L. (1998). Asymmetry of stress–strain curves under tension and compression for NiTi shape memory alloys. Acta Mater..

[18] Mao S.C., Luo J.F., Zhang Z., Wu M.H., Liu Y., Han X.D. (2010). EBSD studies of the stress-induced B2–B19′ martensitic transformation in NiTi tubes under uniaxial tension and compression. Acta Mater..

[19] Reedlunn B., Churchill C.B., Nelson E.E., Shaw J.A., Daly S.H. (2014). Tension, compression, and bending of superelastic shape memory alloy tubes. J. Mech. Phys. Solids.

[20] Bechle N.J., Kyriakides S. (2014). Localization in NiTi tubes under bending. Int. J. Solids Struct..

[21] Jiang D., Kyriakides S., Bechle N.J., Landis C.M. (2017). Bending of pseudoelastic NiTi tubes. Int. J. Solids Struct..

[22] Wagner M.F.-X., Eggeler G. (2006). Stress and strain states in a pseudoelastic wire subjected to bending rotation. Mech. Mater..

[23] He Y.J., Sun Q.P. (2009). Effects of structural and material length scales on stress-induced martensite macro-domain patterns in tube configurations. Int. J. Solids Struct..

[24] He Y.J., Sun Q.P. (2009). Scaling relationship on macroscopic helical domains in NiTi tubes. Int. J. Solids Struct..

[25] Wagner M.F.-X., Dey S., Gugel H., Frenzel J., Somsen C., Eggeler G. (2010). Effect of low-temperature precipitation on the transformation characteristics of Ni-rich NiTi shape memory alloys during thermal cycling. Intermetallics.

[26] Shaw J.A., Kyriakides S. (1995). Thermomechanical aspects of NiTi. J. Mech. Phys. Solids.

[27] Reedlunn B., Daly S., Hector L., Zavattieri P., Shaw J. (2013). Tips and Tricks for Characterizing Shape Memory Wire Part 5: Full-Field Strain Measurement by Digital Image Correlation. Exp. Tech..

[28] Lu H., Cary P.D. (2000). Deformation measurements by digital image correlation: Implementation of a second-order displacement gradient. Exp. Mech..

[29] Delpueyo D., Grediac M., Balandraud X., Badulescu C. (2012). Investigation of martensitic microstructures in a monocrystalline Cu–Al–Be shape memory alloy with the grid method and infrared thermography. Mech. Mater..

[30] Young M.L., Wagner M.F.-X., Frenzel J., Schmahl W.W., Eggeler G. (2010). Phase volume fractions and strain measurements in an ultrafine-grained NiTi shape-memory alloy during tensile loading. Acta Mater..

[31] Khalil-Allafi J., Hasse B., Klönne M., Wagner M., Pirling T., Predki W., Schmahl W.W. (2004). In-situ diffraction investigation of superelastic NiTi shape memory alloys under mechanical stress with neutrons and with synchrotron radiation. Mater. Werkst..

[32] Hsu W.N., Polatidis E., Smid M., Casati N., Petegem S.V., Swygenhoven H.V. (2018). Load path change on superelastic NiTi alloys: In situ synchrotron XRD and SEM DIC. Acta Mater..

[33] Pan B., Qian K., Xie H., Asundi A. (2009). Two-dimensional digital image correlation for in-plane displacement and strain measurement: A review. Meas. Sci. Technol..

[34] GOM (Gesellschaft für Optische Messtechnik mbH) (2010). Software “ARAMIS” v6.3.1 Optical Deformation Analysis.

[35] Pfeiffer S., Frint P., Wagner M.F.-X. (2017). Analysis of the complex stress state during early loading in cylindrical compression-shear specimens. Mater. Sci. Eng..

[36] Elibol C. (2018). Lokalisierungs- und Relaxationsphänomene in Pseudoelastischen und Martensitischen NiTi-Formgedächtnislegierungen. Ph.D. Thesis.

[37] Liu Y., Liu Y., Humbeeck J.V. (1998). Lüders-like deformation associated with martensite reorientation in NiTi. Scr. Mater..

[38] Shaw J.A., Kyriakides S. (1997). On the nucleation and propagation of phase transformation fronts in a NiTi alloy. Acta Mater..

[39] Sun Q.-P., Li Z.-Q. (2002). Phase transformation in superelastic NiTi polycrystalline micro-tubes under tension and torsion––From localization to homogeneous deformation. Int. J. Solids Struct..

[40] Liu Y. (1999). On the nucleation and propagation of stress-induced martensitic transformation in NiTi. Mater. Sci. Eng. A.

[41] Li Z.-Q., Sun Q.-P. (2002). The initiation and growth of macroscopic martensite band in nano-grained NiTi microtube under tension. Int. J. Plast..

[42] Kim J.I., Liu Y., Miyazaki S. (2004). Ageing-induced two-stage R-phase transformation in Ti-50.9at.%Ni. Acta Mater..

[43] Hallai J.F., Kyriakides S. (2013). Underlying material response for Lüders-like instabilities. Int. J. Plast..

[44] Sedmak P., Pilch J., Heller L., Kopecek J., Wright J., Sedlak P., Frost M., Sittner P. (2016). Grain-resolved analysis of localized deformation in nickel-titanium wire under tensile load. Science.

